# RNA-Seq analysis of two brain regions vulnerable to Alzheimer's disease

**DOI:** 10.1186/1753-6561-6-S6-P53

**Published:** 2012-10-01

**Authors:** Xinkun Wang

**Affiliations:** 1 Genomics Facility, The University of Kansas, Lawrence, KS 66047, USA; 2 Higuchi Biosciences Center, The University of Kansas, Lawrence, KS 66047, USA; 3 Department of Pharmacology and Toxicology, The University of Kansas, Lawrence, KS 66047, USA

## Background

Alzheimer's disease (AD), one of the most devastating neurodegenerative diseases, does not affect different brain regions equally. The temporal and frontal lobes are among the brain regions most affected in AD. As the transcriptomic profile of neurons in a certain brain region largely affects their response to pathological conditions like AD, comparative transcriptomic analysis of these susceptible regions can be used to understand why they are particularly vulnerable to the disease. With rapid advances in next-generation sequencing technologies, RNA-Seq, or whole-transcriptome shotgun sequencing, has begun to become a mainstream approach to study brain regions that are affected by neurodegenerative diseases, including AD.

## Materials and methods

In this study, RNA-Seq data that have recently become available [1] from both normally aged and AD brain temporal and frontal lobes (Sequence Read Archive [http://www.ncbi.nlm.nih.gov/sra]; accessions SRX035166, SRX035171, SRX035167 and SRX034874) were analyzed, in order to provide molecular insights into their common vulnerability while accounting for their regional specificities.

## Results

Here we present transcriptomic similarities and differences between the temporal and frontal lobes as detected by RNA-Seq. Interpretation of their similarities helps understand their shared vulnerability to AD. Detection of their differences in both normal aging and AD helps elucidate the progression of this disease in the two different regions.

## Conclusion

The study of AD from the perspective of selective regional vulnerability is the first step towards minimizing its devastating effect on patients through protecting vulnerable brain neurons.
